# CSF GFAP levels in double seronegative neuromyelitis optica spectrum disorder: no evidence of astrocyte damage

**DOI:** 10.1186/s12974-022-02450-w

**Published:** 2022-04-12

**Authors:** Jae-Won Hyun, Yeseul Kim, Ki Hoon Kim, Su-Hyun Kim, Mads Nikolaj Olesen, Nasrin Asgari, Sasitorn Siritho, Friedemann Paul, Ho Jin Kim

**Affiliations:** 1 Department of Neurology, Research Institute and Hospital of National Cancer Center, 323 Ilsan-ro, Ilsandong-gu, Goyang, Korea; 2 Division of Clinical Research, Research Institute and Hospital of National Cancer Center, Goyang, Korea; 3 Department of Regional Health Research & Molecular Medicine, University of Southern Denmark, Odense, Denmark; 4 Department of Clinical Immunology, Odense University Hospital, Odense, Denmark; 5 Department of Neurology, Slagelse Hospital, Slagelse, Denmark; 6 Division of Neurology, Department of Medicine, Siriraj Hospital, Bangkok, Thailand; 7 Bumrungrad International Hospital, Bangkok, Thailand; 8 Department of Neurology, Charité-Universitätsmedizin Berlin, corporate member of Freie Universität Berlin, Humboldt-Universität Zu Berlin, and Berlin Institute of Health, Berlin, Germany; 9 Max Delbrueck Center for Molecular Medicine, Berlin, Germany

**Keywords:** Neuromyelitis optica spectrum disorder, Biomarker, Glial fibrillary acidic protein, Astrocyte, Aquaporin-4, Myelin oligodendrocyte glycoprotein

## Abstract

**Background:**

Despite rigorous confirmation with reliable assays, some individuals showing the neuromyelitis optica spectrum disorder (NMOSD) phenotype remain negative for both aquaporin-4 (AQP4) and myelin oligodendrocyte glycoprotein (MOG) antibodies.

**Objective:**

We aimed to investigate whether double seronegative NMOSD (DN-NMOSD) and NMOSD with AQP4 antibody (AQP4–NMOSD) share the same pathophysiological basis, astrocytopathy, by measurement of cerebrospinal fluid (CSF) glial fibrillary acidic protein (GFAP) levels as a marker of astrocyte damage.

**Methods:**

Seventeen participants who (1) satisfied the 2015 diagnostic criteria for NMOSD, and (2) tested negative for AQP4 and MOG antibodies confirmed with repeated cell-based assays, and (3) had available CSF samples obtained at the point of clinical attacks, were enrolled from 4 medical centers (South Korea, Germany, Thailand, and Denmark). Thirty age-matched participants with AQP4–NMOSD, 17 participants with MOG antibody associated disease (MOGAD), and 15 participants with other neurological disorders (OND) were included as controls. The concentration of CSF GFAP was measured using enzyme-linked immunosorbent assay.

**Results:**

CSF GFAP levels in the DN-NMOSD group were significantly lower than those in the AQP4–NMOSD group (median: 0.49 versus 102.9 ng/mL; *p* < 0.001), but similar to those in the OND (0.25 ng/mL) and MOGAD (0.39 ng/mL) control groups. The majority (90% (27/30)) of participants in the AQP4–NMOSD group showed significantly higher CSF GFAP levels than the highest level measured in the OND group, while no participant in the DN-NMOSD and MOGAD groups did.

**Conclusions:**

These results suggest that DN-NMOSD has a different underlying pathogenesis other than astrocytopathy, distinct from AQP4–NMOSD.

**Supplementary Information:**

The online version contains supplementary material available at 10.1186/s12974-022-02450-w.

## Introduction

Since the discovery of aquaporin-4 (AQP4) antibodies, neuromyelitis optica spectrum disorder (NMOSD) is defined as a neuro-inflammatory disease of the central nervous system (CNS), separate from multiple sclerosis [1]. The majority (up to 90%) of individuals with NMOSD defined by the 2015 revised diagnostic criteria show seropositivity for AQP4 antibody [2, 3]. However, the criteria allow the diagnosis of NMOSD in individuals without AQP4 antibodies [4–6]. A subset of individuals with a NMOSD phenotype, but without AQP4 antibodies, test positive for the myelin oligodendrocyte glycoprotein (MOG) antibody, which primarily targets myelin antigens [7]. Although some clinical features overlap with AQP4 antibody positive NMOSD (AQP4–NMOSD), MOG antibody associated disease (MOGAD) has been recently defined as a distinctive disease entity, with elucidation of plausible different pathophysiology [8]. Despite repeated rigorous antibody measurements with reliable assays, some individuals with NMOSD phenotype remain persistently negative for both AQP4 and MOG antibodies [9–12]. Such double seronegative NMOSD (DN-NMOSD) poses diagnostic and therapeutic challenges in clinical practice and the classification of DN-NMOSD within the neuro-inflammatory diseases of the CNS remains unknown.

AQP4–NMOSD is a primary astrocytopathy mediated by antibodies selectively targeting the water channel protein AQP4, which is abundantly located on astrocytic foot processes in the CNS [1]. Glial fibrillary acidic protein (GFAP), a major constituent of the astrocyte cytoskeleton, can reflect astrocyte injury in AQP4–NMOSD when measured in body fluids, such as cerebrospinal fluid (CSF) [13–15]. This study aimed to investigate whether DN-NMOSD and AQP4–NMOSD share the same pathophysiological basis, astrocytopathy, by comparing the CSF GFAP levels at clinical exacerbation.

## Methods

This international collaborative study enrolled participants from 4 medical centers (South Korea, Germany, Thailand, and Denmark). Participants who (1) satisfied the 2015 diagnostic criteria for NMOSD, [2] (2) were seronegative for both AQP4 and MOG antibodies following rigorous confirmation with reliable assays, and (3) had CSF samples available that were obtained at the point of clinical attack, were included. Seventeen CSF samples were obtained from individuals with DN-NMOSD (11 from South Korea, 2 from Germany, 1 from Thailand, and 3 from Denmark). Thirty age-matched participants with AQP4–NMOSD and 17 participants with MOGAD were included as controls. The MOGAD group included 10 patients who satisfied and 7 who did not satisfy the 2015 criteria for NMOSD. In addition, 15 age-matched controls with other neurological disorders (OND: 4 primary headache, 3 idiopathic sixth cranial neuropathies, 1 compressive myelopathy, 1 sub-acute combined degeneration, 1 hemi-facial spasm, 1 diabetic polyneuropathy, 1 brachial plexopathy, 1 non-specific white matter disease, 1 adrenoleukodystrophy, and 1 suspected motor neuron disease), not expected to present with underlying astrocytopathy, were included. All CSF samples were collected within 1 month of clinical exacerbation and most of the samples were obtained before the initiation of high dose steroid therapy (94% (16/17) in DN-NMOSD, 83% (25/30) in AQP4–NMOSD, and 71% (12/17) in MOGAD).

The serostatus of AQP4 antibodies was rigorously confirmed using two different methods: an in-house live cell-based assay (CBA) conducted at the National Cancer Center (NCC, South Korea) [16], and a commercial CBA (Euroimmun, Luebeck, Germany). The serostatus of anti-MOG antibodies was determined using the in-house CBA at the NCC with live transfected cells with a full-length human MOG [17]. Additional file 1: Table S1 shows titers of AQP4 and MOG antibodies at the time of CSF sampling. Repeated examinations were performed during the course of the disease, at minimum of two different timepoints including at least one acute exacerbation in participants with DN-NMOSD. The seronegative status of the AQP4 and MOG antibodies in the sera, at the same timepoint with the CSF sample collection, 6 participants from Germany, Thailand, and Denmark was double-checked with CBA at each center and NCC. Antibody tests in CSF were also performed to eliminate the possibility of AQP4 and MOG antibodies detectable only in the CSF [18–20]. CSF GFAP concentrations were evaluated in duplicate by an independent investigator who was blinded to the diagnosis using an enzyme-linked immunosorbent assay (ELISA) kit according to the manufacturer’s protocol (BioVendor, Minneapolis, MN, USA).

### Statistical analysis

GFAP levels across groups were compared by the Kruskal–Wallis test and post-hoc analysis was conducted using Dunn’s multiple comparisons test.

## Results

Table 1 shows the demographics of the participants. The mean age at sampling of the participants with DN-NMOSD, AQP4–NMOSD, MOGAD, and OND was 32.3 ± 9.6, 35.2 ± 11.1, 31.2 ± 7.3 and 36.8 ± 7.4 years, respectively. Table 2 demonstrates the clinical and para-clinical features of the participants with DN-NMOSD.

**Table 1:** Demographics of participants

	DN-NMOSD (*n* = 17)	AQP4–NMOSD (*n* = 30)	MOGAD (*n* = 17)	ONDs (*n* = 15)
Age at sampling (years, mean ± SD)	32.3 ± 9.6	35.2 ± 11.1	31.2 ± 7.3	36.8 ± 7.4
Gender (female:male)	10:7	27:3	13:4	8:7
Ethnicity	Korean (11), Thai (1), Caucasian (4), Turkish (1)	Korean (29), Thai (1)	Korean (17)	Korean (15)
Phenotypes met the seronegative NMOSD criteria				
ON + LETM ON + cerebral ON + APS ON + brainstem LETM + brainstem LETM + cerebral Brainstem + cerebral	6 4 1 0 4 1 1	NA	4/10 1/10 0/10 3/10 0/10 1/10 1/10	NA
Attack type at sampling	M (7), B (4), ON (5), Multiple (1)	M (14), B (7), ON (8), Multiple (1)	M (6), B(6), ON (4), Multiple (1)	NA
Presence of maintenance immunosuppressive therapy at sampling	6/17 (35.3%)	14/30 (46.7%)	3/17 (17.6%)	NA
Azathioprine	4	2	2	
Mycophenolate	0	6	0	
Rituximab	1	5	1	
Cyclophosphamide	1	0	0	
Methotrexate	0	1	0	

DN-NMOSD, double seronegative neuromyelitis optica spectrum disorder; AQP4–NMOSD, aquaporin 4 antibody positive neuromyelitis optica spectrum disorder; MOGAD, mog antibody associated disease; ONDs, other neurological disorders; SD, standard deviation; NA, not applicable; ON, optic neuritis; LETM, longitudinally extensive transverse myelitis; APS, area postrema syndrome; M, myelitis; B, brain attack

**Table 2:** Clinical and para-clinical features of double seronegative neuromyelitis optica spectrum disorder (*n* = 17)

CSF-restricted OCB	2/17
CSF pleocytosis (cell > 5/ul)	5/13
MS like lesion on brain MRI	0/17
AQP4–NMOSD like lesion on MRI	
Large, confluent, unilateral, or bilateral subcortical or deep white matter lesion	5/17
Hypothalamic lesion	2/17
Periependymal lesion	5/17
Area postrema lesion	1/17
Brighter spotty lesion on spinal cord [24]	0/11

CSF, cerebrospinal fluid; OCB, oligoclonal band; MS, multiple sclerosis; MRI, magnetic resonance imaging; AQP4–NMOSD, aquaporin-4 antibody positive neuromyelitis optica spectrum disorder

**Fig. 1:**
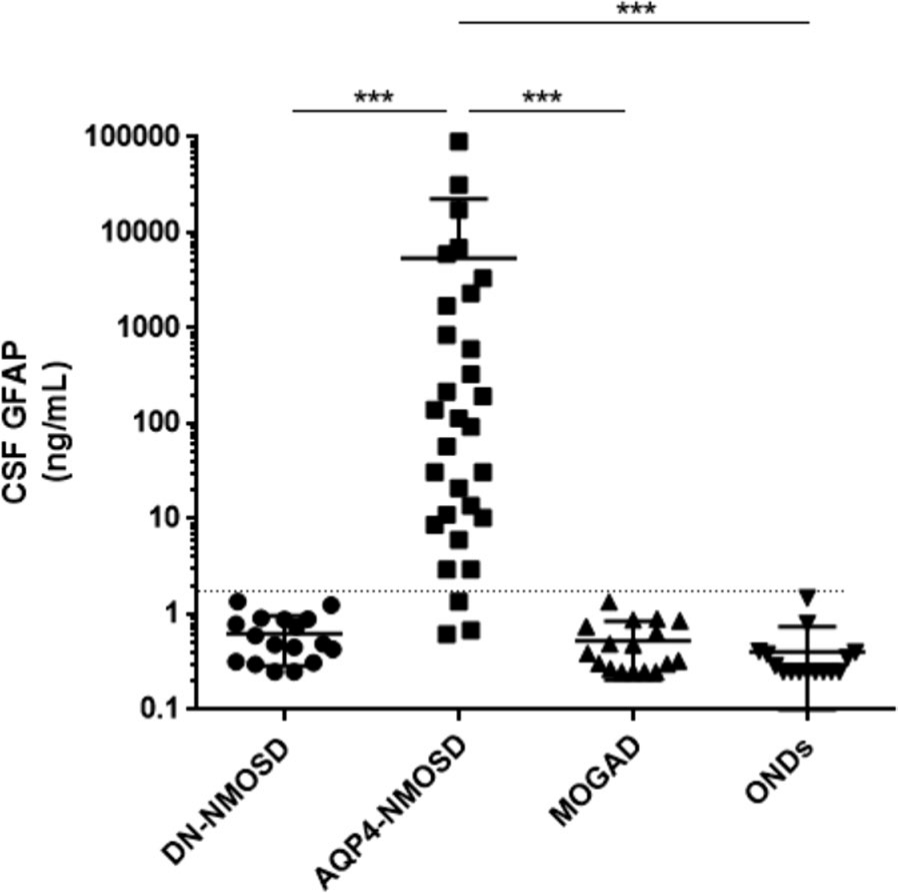
Cerebrospinal fluid (CSF) glial fibrillary acidic protein (GFAP) levels (DN-NMOSD: double seronegative neuromyelitis optica spectrum disorder, AQP4–NMOSD: anti-aquaporin-4 antibody positive neuromyelitis optica spectrum disorder, MOGAD: anti-myelin oligodendrocyte glycoprotein antibody associated diseases, ONDs: other neurological disorders; dot line: the highest measured GFAP level in ONDs, ***p < 0.0001)

As shown in Fig. 1, the concentration of CSF GFAP in the DN-NMOSD group was significantly lower than that in the AQP4–NMOSD group (median: 0.49 (range 0.25–1.36) versus 102.9 (range 0.62–90,242.6) ng/mL; *p* < 0.001), but similar to that in the OND (0.25 (0.25–1.48) ng/mL) and MOGAD (0.39 (0.25–1.35) ng/mL) control groups.

None of the DN-NMOSD and MOGAD groups showed higher CSF GFAP levels than the highest measured CSF GFAP level in participants with ONDs (primary headache, 1.48 ng/mL). In contrast, the CSF GFAP levels of most participants (27/30, 90%) in the AQP4–NMOSD group were higher than the highest measured CSF GFAP level in the OND group, and the remaining 3 participants with AQP4–NMOSD experienced relatively mild clinical symptoms (2 with optic neuritis and 1 with sensory-only partial myelitis).

## Discussion

The CSF GFAP levels in the rigorously confirmed DN-NMOSD group were significantly lower than those in the AQP4–NMOSD group, but did not differ from those in the OND or MOGAD group. Consistent with previous results, [12–15] CSF GFAP levels in participants with AQP4–NMOSD were significantly higher than those in participants with ONDs or MOGAD. These findings suggest a discriminative underlying pathophysiology in DN-NMOSD other than astrocytopathy, distinguishing it from AQP4–NMOSD.

It is important to confirm the truly negative status of AQP4 and MOG antibodies to facilitate a fair comparison between DN-NMOSD and AQP4–NMOSD. Therefore, repeated tests using reliable CBAs were performed at least two different timepoints, particularly in the clinical exacerbation, to detect the positive conversion of AQP4 and MOG antibodies during subsequent clinical relapse [21]. At least two different assays with high sensitivity and specificity were used [16, 17], in addition to CSF assays, because antibodies in some cases may be only detectable in CSF but not in serum [18–20]. A previous study reported that CSF GFAP levels in 2 of 3 participants with DN-NMOSD were elevated above those in non-neurological controls (highest level: 2.3 ng/mL) and increased over the median CSF GFAP level (5.40 ng/mL) of AQP4–NMOSD [12]; whereas no elevation in CSF GFAP levels was observed in 17 participants with DN-NMOSD relative to those in controls with ONDs (highest level: 1.48 ng/mL) in current study. The reason for this discrepancy is yet to be uncovered. Given the possible heterogeneity of DN-NMOSD, there would be a subset of patients with astrocytopathy due to yet unidentified autoantibodies or other causes in 3 DN-NMOSD patients of previous study, but not in 17 patients of current study. Alternatively, it might simply result from the difference in the confirmation of double negativity against AQP4 and MOG antibodies. The median CSF GFAP level of controls with ONDs (0.25 ng/mL) in the current study was comparable to that of previous studies (median 0.5 ng/mL in non-neurological controls [12] and mean 0.6–0.7 ng/mL in controls with ONDs [13, 14]).

## Conclusions

The current evidence of the pathophysiological distinction between DN-NMOSD and AQP4–NMOSD raises an issue whether these two potentially different diseases should be within the same umbrella of NMOSD. Two recent clinical trials that applied the 2015 NMOSD criteria revealed that the efficacy of therapeutic agents for AQP4-negative NMOSD was unclear compared to that for AQP4-positive NMOSD [22, 23]. To establish adequate therapeutic strategies on the basis of appropriate pathophysiology, further investigations to identify other potential immunological target(s) specific to DN-NMOSD are warranted.

## Supplementary Information

**Additional file 1. Supplementary table 1.** The titers of AQP4 and MOG antibodies.

## Data Availability

Anonymized data not published within this article will be made available upon request from any appropriately qualified investigator.

## Abbreviations

AQP4: Aquaporin 4
NMOSD: Neuromyelitis optica spectrum disorder
CNS: Central nervous system
MOG: Myelin oligodendrocyte glycoprotein
AQP4–NMOSD: AQP4 antibody positive NMOSD
MOGAD: MOG antibody associated disease
DN-NMOSD: Double seronegative NMOSD
GFAP: Glial fibrillary acidic protein
CSF: Cerebrospinal fluid
OND: Other neurological disorder
CBA: Cell based assay
