# Supplementary material for: CSF GFAP levels in double seronegative neuromyelitis optica spectrum disorder: no evidence of astrocyte damage
Source: J Neuroinflammation. 2022 Apr 12;19:86. doi: 10.1186/s12974-022-02450-w (PMC9006458; doi:10.1186/s12974-022-02450-w)
Supplement: Supplementary file 1 — Additional file 1. Supplementary table 1. The titers of AQP4 and MOG antibodies. [file 12974_2022_2450_MOESM1_ESM.docx]

**Supplementary table 1. The titers of AQP4 and MOG antibodies**

| AQP4 antibody titers in AQP4-NMOSD | Number of AQP4-NMOSD participants (total n = 30) | MOG antibody titers in MOGAD | Number of MOGAD participants  (total n = 17) |
| --- | --- | --- | --- |
| 1+ | 14 | 1+ | 7 |
| 2+ | 5 | 2+ | 7 |
| 3+ | 6 | 3+ | 2 |
| 4+ | 5 | 4+ | 1 |

Abbreviations: AQP4, aquaporin-4; MOG, myelin oligodendrocyte glycoprotein; AQP4-NMOSD, aquaporin-4 antibody seropositive neuromyelitis optica spectrum disorder; MOGAD, myelin oligodendrocyte glycoprotein antibody associated diseases
